# The effect of bergamot polyphenolic fraction on lipid transfer protein system and vascular oxidative stress in a rat model of hyperlipemia

**DOI:** 10.1186/s12944-019-1061-0

**Published:** 2019-05-17

**Authors:** Vincenzo Musolino, Micaela Gliozzi, Saverio Nucera, Cristina Carresi, Jessica Maiuolo, Rocco Mollace, Sara Paone, Francesca Bosco, Federica Scarano, Miriam Scicchitano, Stefano Ruga, Maria Caterina Zito, Carmen Colica, Roberta Macrì, Ernesto Palma, Salvatore Ragusa, Carolina Muscoli, Vincenzo Mollace

**Affiliations:** 1 Institute of Research for Food Safety & Health (IRC-FSH), Department of Health Sciences, University “Magna Graecia” of Catanzaro, Viale Europa, Loc. Germaneto, 88100 Catanzaro, Italy; 2 Nutramed S.c.a.r.l., Complesso Ninì Barbieri, 88021 Roccelletta di Borgia Catanzaro, Italy; 3 Department of Health Sciences, University “Magna Graecia” of Catanzaro, Catanzaro, Italy; 4 San Raffaele IRCCS Pisana, Rome, Italy

**Keywords:** Bergamot polyphenolic fraction, Lipid transfer protein system, Hyperlipidaemia, Oxidative stress

## Abstract

**Background:**

Experimental and epidemiological studies show that bergamot polyphenolic fraction (BPF) ameliorates the serum lipemic profile, normalizes blood pressure and improves non alcoholic fatty liver disease in patients suffering from metabolic syndrome. Despite this evidence, the molecular mechanisms responsible for these beneficial effects remain unclear. The aim of our study is to clarify the effects of BPF on the lipoprotein assembly and to identify oxidative stress biomarkers correlating hyperlipidaemia and BPF-induced metabolic changes.

**Methods:**

Male Wistar rats (180–200 g) were randomly assigned to receive a standard diet, a hypercholesterolemic diet or a hypercholesterolemic diet+BPF (20 mg/Kg/rat daily, gavage), respectively, for 90 days. Total cholesterol (tChol), high density lipoprotein cholesterol (HDL-C), low density lipoprotein cholesterol (LDL-C), triglycerides (TG) and fasting plasma glucose were evaluated at the baseline as well as at the end of the treatment. To assess the effect of BPF on the Lipid Transfer Protein System, detection of ACAT, LCAT, CETP, PON1, Apo A1 and Apo B have also been carried out. Finally, the lipid peroxidation biomarker (TBARS) and oxyLDL were also measured.

**Results:**

BPF prevented tChol, LDL-C, TG and fasting plasma glucose enhancement and improved HDL-C. Treatment of hyperlipæmic rats with BPF significantly restored altered the serum concentration of lipemic biomarkers and the activity of ACAT, LCAT, CETP and PON1, an effect accompanied by the concomitant normalization of Apo A1 and APO B levels. In addition, TBARS levels were reduced significantly by the treatment with BPF.

**Conclusions:**

BPF prevents diet-induced alteration of the lipid profile in rats, counteracting oxidative stress and improving the dysregulation of the Lipid Transfer Protein System. These data add new insights into the molecular mechanisms underlying the beneficial role of BPF in the therapy of hyperlipidaemia, thus suggesting a novel approach in the prevention of cardiovascular disease.

## Background

Evidence has been accumulated demonstrating that bergamot polyphenolic fraction (BPF) is able to produce hypolipemic effect accompanied by improvement of endothelial function and reduction of cardiometabolic risk. This has been assessed in both animal models of hyperlipemia as well as in patients [1].

In particular, BPF supplementation has been shown to reduce serum cholesterol, LDL and triglycerides, leading to significant improvement of overall parameters of metabolic balance, an effect which includes glycemic control and weight reduction in subjects suffering from Metabolic Syndrome (MS). Several mechanisms have been suggested to explain the ameliorated metabolic balance observed in animals and humans supplementing with Bergamot polyphenols [1]. Indeed, a significant reduction of cholesterol absorption has been demonstrated to occur following supplementation with BPF, an effect which seems to be related to the inhibition of pancreatic cholesterol ester hydrolase (pCEH); an enzyme which contributes in the cholesterol absorption. In particular, fecal sterol excretion was found to be increased in hyperlipidemic rats, an effect enhanced by bergamot juice and BPF [2, 3]. The increase in the excretion of bile acids seems to activate cholesterol 7R-hydroxylase, enhancing the conversion of liver cholesterol to bile acids for excretion.

This leads to a decrease in hepatic cholesterol content, which in turn stimulates LDL receptor expression and lowers blood cholesterol levels. In addition, the traffic of lipoproteins in the liver is ameliorated by BPF, thereby contributing in the relevant hypolipemic response found in patients with MS and liver dysfunction. In particular, a significant reduction of small dense atherogenic LDL lipoproteins was found in patients with MS, an effect associated with an increase of larger size lipoproteins, thereby leading to a better lipemic profile. In addition, both hystopathological and serum biomarkers of non-alcoholic liver disease (NAFLD) were normalized by BPF, thus suggesting a clear correlation between the effect of BPF and the improvement of biochemical modulation occurring in liver tissues [4]. Moreover, recent data showed that BPF directly inhibits the endogenous biosynthesis of cholesterol, acting directly on the rate limiting enzyme hydroxy-methyl-glutaryl CoA reductase (HMGCoA reductase) [5]. In particular, BPF has been shown to contain large amounts of glycosylated polyphenols (in particular bruteridine and melitidine), which have been shown to possess statin-like activity, thereby inhibiting HMGCoA reductase [6].

Finally, we have demonstrated that BPF modulates AMP (adenosine monophosphate)-activated protein kinase (AMPK) which plays a key role in the regulation of the metabolic pathways involved in ATP production in mammalian cells [7]. On the other hand, AMPK plays a central role in the integrated modulation of both lipidic and glycidic metabolism in the liver, while dysregulation of AMPK has been associated with fat accumulation and liver dysfunction in subjects with hyperlipemia [8]. Thus BPF, due to this effect in modulating AMPK, contributes to a better lipemic profile.

Although clear evidence accounts for a beneficial role of supplementation with BPF in hyperlipemic patients, the molecular mechanism contributing to this effect still needs to be better clarified.

In particular, the mechanism leading to BPF-related conversion of cholesterol to cholesteryl esters and the subsequent lipoprotein assembly represents an effect mostly due to the activity of thr Lipid Transfer Protein system. On the other hand, the action of bergamot polyphenols on folding functional HDLs and enhancing lipid exchange among different lipoproteins, regarding the effect of BPF on HDL levels in hyperlipemic patients, needs to be further explored.

The present experiments have been performed in order to investigate on the effect of BPF in the biomarkers of lipoprotein assembly and functional regulation in rats fed a hyperlipemic diet. In addition, the possible correlation between metabolic changes induced by BPF in hyperlipemic rats and oxidative stress biomarkers have also been explored.

## Methods

### Study design

BPF, as previously prepared and characterized by polyphenol content [1, 5] was kindly provided by Herbal and Antioxidant Derivatives S.r.l. (Polistena, RC, Italy).

All the experimental procedures were performed according to protocols approved by the Animal Care of University Magna Graecia of Catanzaro, in accordance with the European Commission guidelines (Directive 2010/63/EU) for the animals used for scientific purposes.

Male Wistar rats (Charles River, Italy), weighing 180–200 g, were used for the experiments. The animals were kept under stable and controlled conditions (temperature, 22 °C; humidity, 60%) with water ad libitum.

The rats were divided into four groups of 10 animals each:

- Group I was kept on a standard diet (Envigo) for 90 days.
- Group II received the hypercholesterolemic diet for 90 days.
- Groups III received the hypercholesterolemic diet for 90 days; from the 1st to the 90th day, each rat was administered with BPF (20 mg/kg/daily) by gavage.

During the experiment, animals were weighed weekly, and 24 h food consumption was recorded daily. On day 90, rats were individually housed in metabolic cages. At the end of the study, the animals were fasted overnight. For the sacrifice, rats were sedated with 30 mg/Kg of tiletamine-zolazepam (Zoletil. 50/50 mg/ml, Virbac, Milan, Italy) and then exposed to a constant flow rate of 5% of isoflurane (Iso-Vet, Piramal Healthcare UK Limited, United Kingdom). Isoflurane exposure was continued aproximatly until one minute after breathing stop. Euthanasia was confirmed by cervical dislocation. Blood samples were collected via cardiac puncture from the heart of euthanized rats, serum was separated and stored at − 20 °C until analyzed. In addition, samples (10 mg) of liver tissues were collected after sacrifice.

The effects of BPF on total serum cholesterol (tChol), high density lipoprotein cholesterol (HDL-C), low density lipoprotein cholesterol (LDL-C), triglycerides (TG) and fasting plasma glucose were evaluated at baseline as well as at the end of treatment (90 days) in Wistar rats fed a standard diet (control group) or hypercholesterolemic diet composed of a standard diet (Envigo), supplemented with cholesterol 2% (Sigma-Aldrich, pur. 95%), 0.2% cholic acid (min. 98%, Sigma) and 4.8% palm oil. Moreover, to assess the effect of treatment on Lipid Transfer Protein System, detection of acetyl-Coenzyme A acetyltransferase (ACAT), Lecithin Cholesterol Acyltransferase (LCAT), Cholesteryl ester transfer protein (CETP), paraxonoase 1(PON1), Apolipoproteina **A1 (**Apo A1) and Apolipoproteina B (Apo B) have also been carried out at baseline and after 90 days of treatment with BPF or placebo in normal as well as in hyperlipemic rats. Finally, measurements of the lipid peroxidation biomarker (TBARS) and oxyLDL were carried out at baseline and after the 90 day treatment schedule with placebo or BPF to assess the effect of dietary supplementation on oxidative stress biomarkers.

### Serum lipid measurements

Analysis were performed at baseline and after 90 days in each group. For the baseline measurement, approximately 1 mL blood was collected from lateral tail vein into untreated Eppendorf tubes and centrifuged for 15 min at 1500 g at 4 °C. The serum was then collected and stored at − 80 °C for later analysis. The day of the sacrifice, blood was collected via cardiac puncture from the heart of euthanized mice. A volume of around 1 mL of whole blood was collected into untreated Eppendorf tubes then centrifuged for 15 min at 1500 g at 4 °C. The serum was then collected and stored at − 80 °C for later analysis. tChol, LDL-C, HDL-C, tryglycerides and glucose were evaluated in serum samples, using an automatic chemistry analizer, XL-640 (Erba Mannhein) and the following Erba liquid stable reagents: Total Cholesterol (CHOL 440, XSYS0009), Low-density lipoproteins (LDL C 80, XSYS0044), Triglycerides (TG 440, XSYS0041), High-density lipoprotein (HDL C160, XSYS0043) Glucose (GLU 440, XSYS0012). Under the same treatment schedule, Serum ApoAI and ApoB levels were detected through the immunoturbidimetric immunoassay using a commercial kit (Kamiya Biomedical - Fisher Scientific, Hampton, USA). Briefly, the Cobas-Bio centrifugal analyzer and commercially available antisera (K-ASSAY; APO-AI cat. N. KAI-002; APO-B cat. N. KAI-004) and calibrators (K-ASSAY; APO AI/B Calibrator Cat. No. KAI-008C) were used. The ApoA and ApoB antibodies interact with the Apo A and Apo B in the serum, forming immune complexes. The immune complexes can produce an enhancement in light scattering, which can be measured spectrophotometrically at 600 nm. Since the increase in turbidity is proportional to the amount of Apo A or Apo B in the sample, the apolipoprotein A and B concentration can be determined by measuring this increase in turbidity.

### ACAT assays

ACAT activity was assayed in liver tissues of rats at day 90, after sacrifice.

Briefly, flash frozen liver samples from each group were used to determine ACAT activity. Microsomes were isolated from the liver samples as previously described [9]. Microsomal protein concentration was measured by the method of Lowry et al. A 50 μg aliquot of microsomal protein was used to determine ACAT activity as previously described. In summary, microsomal protein, 1 mg of BSA, and 50 nmol of free cholesterol in 45% (w/v) β-cyclodextrin were mixed together and subsequently incubated for 30 min at 37 °C. Then, 30 nmol of [^14^C]oleoyl-CoA was added to the mix and incubated for 10 min. The reaction was halted by the addition of 2:1 chloroform-methanol. After the phases separated, the organic phase was removed and applied to a TLC plate. The CE band was scraped, suspended in scintillation fluid, and counted for ^14^C radioactivity.

ACAT activity was specifically determined by comparing the results of BPF on total ACAT activity to those obtained with the vehicle-treated group.

### Detection of CETP and LCAT

A serum CETP assay kit (BioVision Inc., CA, USA), using a donor molecule containing a fluorescent self-quenched neutral lipid was transfererred to an acceptor molecule resulting in fluorescence (Excitation: 465 mn; Emission: 535 nm). The serum LCAT assay kit (ELISA, Life Inc., Wuhan, China) was used. The kit is a sandwich enzyme immunoassay for the in vitro quantitative measurement of rat’s LCAT in serum. The microtiter plate provided in this kit had been pre-coated with an antibody specific to LCAT. Standards or samples are then added to the appropriate microtiter plate wells with a biotin-conjugated polyclonal antibody preparation specific for LCAT. Next, avidin, in conjunction with horse radish peroxidase (HRP), was added to each well. Only those wells that contained LCAT, biotin-conjugated antibody and enzyme-conjugated avidin exhibited a change in color. The enzyme-substrate reaction was terminated by the addition of a sulfuric acid, and the color change was measured spectrophotometrically at a wavelength of 450 nm ± 10 nm. The LCAT concentration in the samples was then determined by comparing the O.D. of the samples to the standard curve.

### Analysis of paraoxonase (PON1) activity

PON1 activity was measured in the serum of the hyperlipemic rats. Briefly, we set up the following enzymatic reaction using paraoxon (O,O-diethyl-O-p-nitrophenylphosphate) as a substrate and the generation of 4-nitrophenol, was followed spectrophotometrically: 50 μL serum was dissolved in 1 mL Tris/HCl buffer (100 mmol/L, pH 5 8.0) containing 2 mmol/L CaCl_2_ and 5.5 mmol/L paraoxon. The absorbance was measured at 412 nm (25 °C), using the Hewlett-Packard 8453 ultraviolet-visible spectrophotometer (Hewlett-Packard, Palo Alto, Calif). Enzyme activity was measured using the molar extinction coefficient 17,100 mol^− 1^ cm^− 1^. One unit of PON1 activity was defined as 1 nmol of 4-nitrophenol formed per minute.

### Oxidative stress detection

Serum lipid peroxidation products were assessed using a thiobarbituric acid reactive substances (TBARS) assay. After precipitation of non-lipid TBARS with ice-cold 10% (w/v) trichloroacetic acid, 0.33% TBA (w/v) was added to the supernatant, boiled for 10 min, and fluorescence read at 530 nm excitation and 550 emission on a fluorescence microplate reader (BioTek, Winooski, VT).

### Measurement of oxidized low density lipoproteins (oxy-LDL)

Oxy-LDL was measured using the commercially available Mercodia Oxy-LDL Competitive ELISA kit (Mercodia AB, Sylveniusgatan 8A, SE-754 50 Uppsala, Sweden), intended to be used for the in vitro quantitative determination of Oxy-LDL in human serum or plasma. The Mercodia Oxy-LDL ELISA kits uses a monoclonal antibody, 4E6, which is specific for oxidatively modified LDL. The 4E6 antibody is directed against a conformational epitope in the apoB-100 moiety of LDL that is generated as a consequence of aldehyde substitution of the lysine residues of apoB-100.

The principle of the procedure is based on the fact that Oxy-LDL in the sample competes with a fixed amount of Oxy-LDL bound to the microtiter well to allow the binding to the biotin-labeled specific antibodies 4E6. After a washing step that removes un-reactive sample components, the biotin-labeled antibody bound to the well is detected by HRP-conjugated streptavidin. After a second incubation and an additional washing step, the bound conjugate is detected by reaction with 3,3′,5,5′-tetramethylbenzidine (TMB). The reaction is stopped by adding acid to give a colorimetric endpoint that is read spectrophotometrically.

Concentrations or activities were calculated based on standard curves. Control samples were run with each assay for quality control purposes.

Laboratory analyses were performed blinded with respect to the assigned treatment.

### Statistics

Data were expressed as mean ± standard error (SEM) and statistically evaluated for differences using *two tailed-t* test. A *p* value of < 0.05 was considered significant.

## Results

In animals fed a hypercholesterolemic diet for 90 consecutive days, an elevation of tChol, LDL-C, tryglicerides and fasting glucose was found compared to baseline values (*n* = 10; Table 1). In contrast, diet-induced hyperlipemia produced a reduction of HDL-C. In rats receiving a standard diet, no changes were observed in any of the lipemic and glycemic biomarkers measured at baseline and after 90 days (*n* = 10; Table 1). Administration of BPF (20 mg/kg/daily; n = 10) for 90 days in diet-induced hypercholesterolemic rats produced significant reduction in tChol, cLDL, tryglycerides and glucose, an effect accompanied by significant elevation of cHDL (Table 1) compared to hyperlipemic animals. All the animals among different groups survived after 90 days. No significant difference in weekly mean body weight, in BPF-treated group versus hyperlipidemic controls was found and no reduction in 24 h food consumption was observed. Moreover, BPF treatment did not produce any change in white and red blood cell counts nor were any changes observed in functional parameters of liver and renal function (data not shown).

**Table 1:** The effect of BPF on serum lipids, Apo A1 and Apo B, LCAT, CETP and PON1 activity in rats fed normal diet (normolipidemia) of after 90 days of hyperlipemic diet (Hyperlipidemia) compared to baseline levels

Parameter	Baseline	Normo- lipidemia	Baseline	Hyper- lipidemia	Baseline	Hyper- lipidemia + BPF
Number	10	10	10	10	10	10
tChol (mmol/L)	152 + 9	154 ± 10	153 + 8	210 ± 9*	153 + 9	165 ± 10^§^
Triglyceride (mmol/L)	152 + 7	151 ± 8	153 + 10	190 ± 9*	150 + 8	160 ± 9^§^
HDL-C (mmol/L)	50 + 5	50 ± 4	48 + 5	41 ± 6*	48 + 6	49 ± 4^§^
LDL-C (mmol/L)	72 + 5	73 ± 5	74 + 6	131 ± 6*	75 + 6	84 ± 7^§^
Apo A1 (g/L)	1.57 + 0.15	1.56 ± 0.14	1.58 + 0.13	1.32 ± 0.11*	1.55 + 0.15	1.48 ± 0.12^§^
ApoB (g/L)	0,62 + 0.2	0.60 ± 0.2	0.62 + 0.2	1.04 ± 0,3*	0.61 + 0.3	0.82 ± 0.2^§^
LCAT (pg/mL)	45 + 12	44 ± 11	43 + 10	32 ± 9*	44 + 13	49 ± 11^§^
CETP (pmol/ml/h)	135 + 12	135 ± 10	133 + 10	152 ± 11*	135 + 11	138 ± 13^§^
PON1	81 + 6	83 + 5	84 + 6	56 + 6*	82 + 7	78 + 5^§^

Data are expressed as Mean ± SEM

*: *p* < 0.05 baseline vs hyperlipidemia

^§^ p < 0.05 hyperlipemia vs BPF-treated rats

**Fig. 1:**
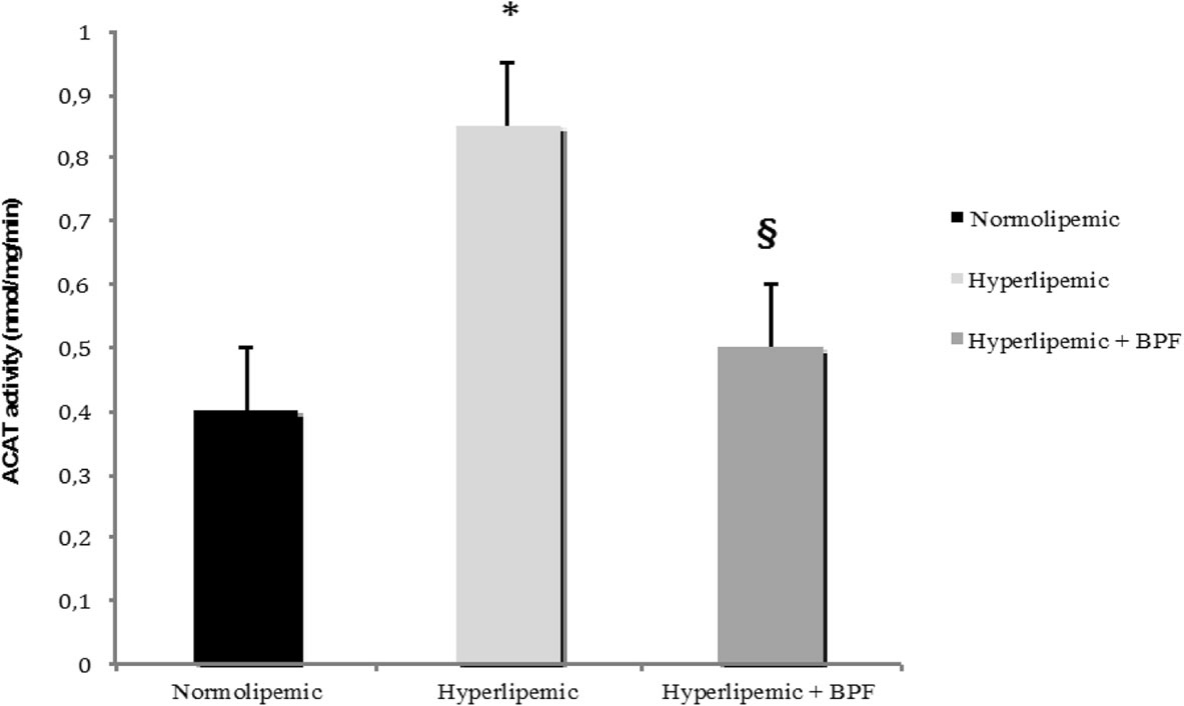
Effect of BPF on ACAT activity in the liver of hyperlipemic rats. ACAT activity was assayed in microsomes isolated from liver samples of normolipemic as well as in hyperlipemic rats either untreated or treated with BPF (20 mg/Kg daily × 90 days). Activity is reported as nmol [^14^C] oleoyl-CoA per mg microsomal protein per min. Data are expressed as Mean ± SEM. *: *p* < 0.05 vs normolipemic. §: *p* < 0.05 vs hyperlipemic

The effect of BPF in counteracting alteration of the lipemic serum and glycemic profile induced by diet-related hyperlipemia was accompanied by significant improvement of Lipid Transport Protein System as detected by measurements of ACAT, LCAT, CETP and PON1 (Table 1, Fig. 1). In particular, induction of hyperlipemia in rats fed a hyperlipemic rich diet for 90 consecutive days was accompanied by a significant increase of ACAT and CETP, this effect being associated with a significant reduction of LCAT and PON1 compared to normolipemic animals. In addition, concomitant changes induced by hyperlipemia were seen in APO A1 and Apo B. Indeed, in hypercholesterolemic rats, decreased Apo A1 levels were accompanied by an elevated concentration of Apo B, thus suggesting that diet-induced hyperlipemia in rats is associated with imbalanced regulation of Lipid Transfer Protein System (Table 1).

Treatment of hyperlipemic rats with BPF, significantly restored altered serum concentration of lipemic biomarkers, serum concentration of LCAT as well as the activity of ACAT, PON1 and the transfer activity of CETP, an effect accompanied by concomitant normalization of Apo A1 and APO B levels (Table 1; Fig. 1).

**Fig. 2:**
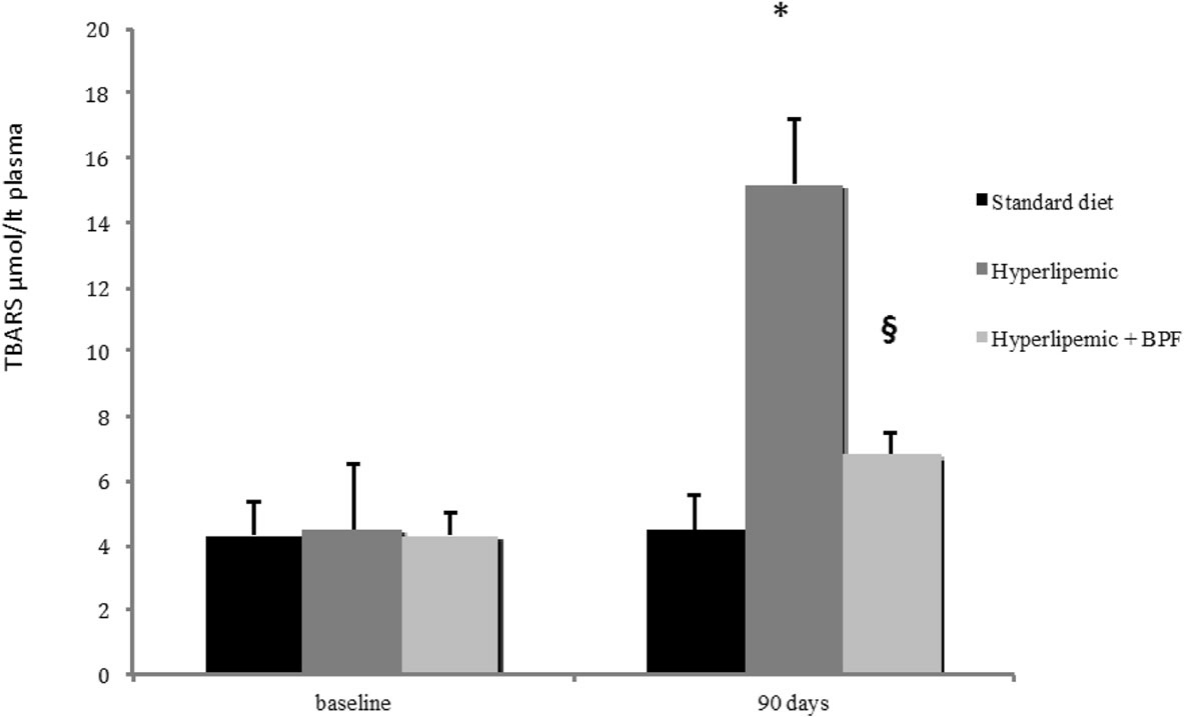
Effect of BPF on hypercholesterolemic diet-induced oxidative stress. Serum lipid peroxidation products were assessed by TBARS assay in normolipemic as well as in hyperlipemic rats either untreated or treated with BPF (20 mg/Kg daily × 90 days). Concentration of TBARS (μmol/Lt plasma), which was found increased in hyperlipemic rats, was reduced significantly by treatment with BPF. Data are expressed as Mean ± SEM. *: *p* < 0.05 vs normolipemic. §: *p* < 0.05 vs hyperlipemic

**Fig. 3:**
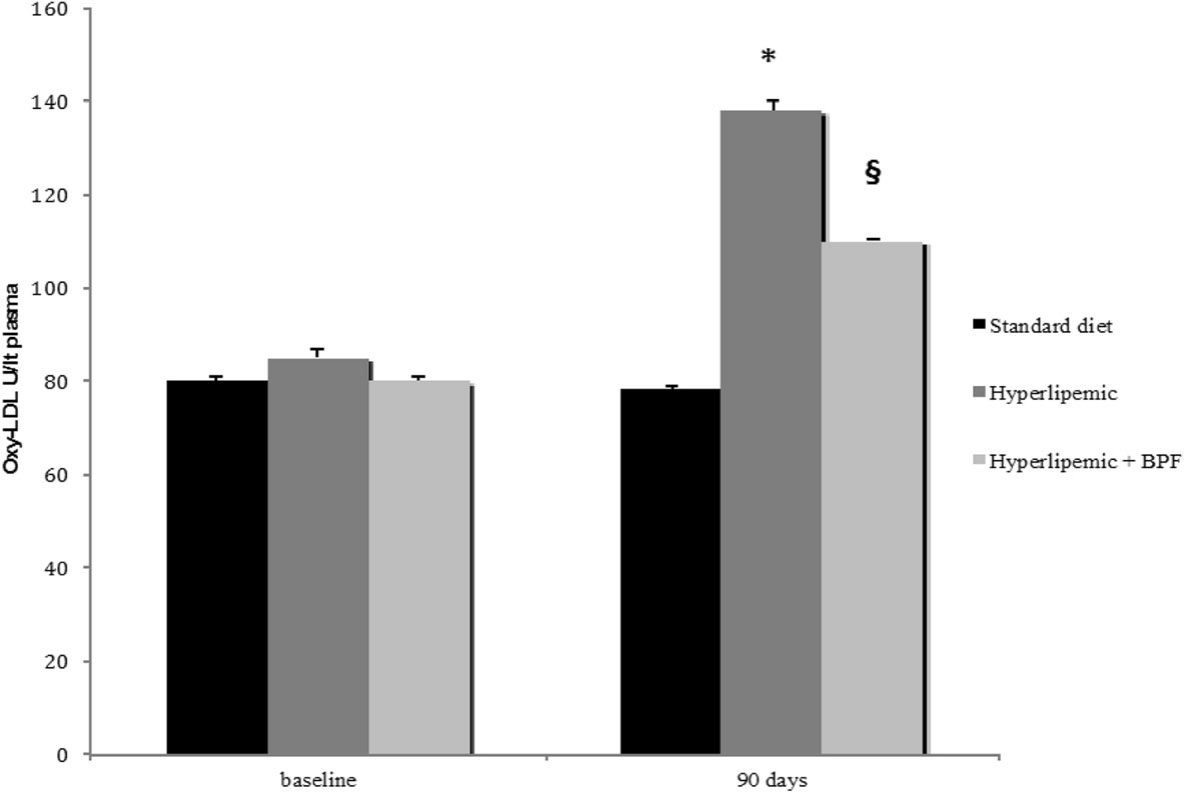
Effect of BPF on Oxy-LDL concentrations in hyperlipemic rats. Oxy-LDL concentrations were measured by ELISA kit in rats blood plasma. Oxy-LDL concentrations were significantly increased by hyperlipemic diet. This effect was significantly antagonized by treatment of animals with BPF (20 mg/Kg daily × 90 days). Data are expressed as Mean ± SEM. *: *p* < 0.05 vs normolipemic. §: *p* < 0.05 vs hyperlipemic

The effect of BPF was accompanied by a significant reduction of oxidative stress induced by a hypercholesterolemic diet in rats. In fact, concentration of TBARS, a biomarker of lipid peroxidation, which was found to be increased in hypelipemic rats, was reduced significantly by treatment with BPF (Fig. 2). This effect also displayed a significant change in oxyLDL content, a well recognized predictor of cardiometabolic risk. Indeed, oxyLDL concentrations were significantly increased by diet induced hyperlipemia in rats. This effect was antagonized by treatment of animals with BPF (Fig. 3), thus suggesting that BPF leads to significant beneficial response on both oxidative stress and cardiometabolic risk as well as the improvement seen in the Lipid Transfer Protein System.

## Discussion

The present data show, for the first time, that the hypolipemic properties of BPF, a polyphenol-rich extract deriving from *Citrus bergamia* Risso & Poiteau, are associated with the modulation of Lipid Trasfer Proteins and this accounts for the role of dietary supplementation of BPF in preventing the development of atherosclerosis in a rat model of hyperlipemia. This effect is strongly correlated to the antioxidant properties of BPF [10] and is in accordance with previous data collected both in animal models of hyperlipemia as well as in patients [1–5].

The role of Lipid Transfer Proteins in the regulation of cholesterol transport and metabolism has widely been assessed in the last few decades [9]. Indeed, it has been clarified that lipid transfer proteins (LTPs) can represent an additional mechanism, characterized by different reactions of cholesterol esterification, able to mediate traffic between organelles in a vesicular-independent manner by removing lipids from the membrane, transporting lipids into the cytosol, and re-inserting lipids into a different membrane [11]. Moreover, these enzymes contribute to the modification of plasma lipoprotein particle composition in order to transfer cholesterol throughout tissues to maintain its homeostasis. As a consequence, the impairment of this enzymatic pattern represents a very important risk factor for the onset of metabolic and cardiovascular diseases [10]. Our results show an elevation of tChol, LDL-C, triglycerides and fasting glucose in animals fed a hypercholesterolemic diet as well as elevations of ACAT and CETP. Under physiological conditions, ACAT catalyzes the esterification of cholesterol in the intestine prior to its incorporation into chylomicrons for circulation, whereas CEPT mediates the transfer of cholesteryl ester from HDL to LDL or VLDL in exchange for TAG [12] and their dysfunction contributes to the development of hypercholesterolemia. BPF was able to reduce total cholesterol and LDL cholesterol produced by dietary supplementation in hyperlipemic rats and to increase HDL content, an effect accompanied by a reduction of ACAT activity. The transport of cholesterol from peripheral tissues to the liver by HDL is strongly regulated by LCAT, a sugar-rich glycoprotein which catalyzes the biosynthesis of the most relevant fraction of cholesteryl ester in the plasma. In contrast with ACAT-deficient mice, the animals in which LCAT expression was suppressed showed an increased cholesterol oxidation, reduced HDL and sustained atherosclerosis development [13]. BPF enhances LCAT, suggesting that hyperlipidemia is able to affect the physiological function of these LTPs. Apo A1 represents the major activator of LCAT and it has been demonstrated that endogenous as well as dietary factors modulating Apo A1 expression lead to LCAT changes and to the development of atherogenesis during hyperlipemic disease states [14]. BPF was able to normalize the levels of Apo A1, further confirming its beneficial effect against hyperlipidemia.

Previous results showed that BPF can determine an improvement as well as a re-arrangement of lipoprotein particle profile, reducing small LDL atherogenic particles and improving large anti-atherogenic HDL in patients with elevated cardiometabolic risk. Our results extend this evidence as we have demonstrated that BPF is able to modulate CEPT, an enzyme involved in the exchange of cholesteryl ester between lipoproteins; in particular, it catalyzes the transfer of cholesteryl ester from HDL to triglyceride rich lipoproteins and to LDL. The discovery of CETP polymorphism associated with the development of Coronary Artery Disease due to accelerated development of atherosclerotic process and the generation of dysfunctional HDL has shed new light on the potential role of CETP modulation in hyperlipemic animals and humans [15]. From this perspective, the use of BPF might also contribute to the prevention of Coronary Artery Disease acting on CEPT.

Finally, a significant effect of BPF in protecting LDLs from oxidative processes has been shown in the reduction of PON1 paraxonase activity. In vitro and in vivo studies have demonstrated that PON1 can lead to hydrolysis of oxidized fatty acids deriving from phospholipids, cholesteryl ester and triglycerides hydroperoxides which are known to be involved in the atherogenesis process [16], suggesting that BPF also guards against oxidative damage through the normalization of PON1 activity.

In addition, we hypothesize that the beneficial effect of PON1 modulation by BPF might also be exerted by the inhibition of NF-kB activation, Specificity Protein 1 (SP1) and Sterol Regulatory Element-Binding Protein 2 (SREBP2), in agreement with in vitro and in vivo studies showing that the supplementation with dietary polyphenols, acting on these protein targets, are able to mediate the anti-atherogenic response in order to counteract the cardiometabolic risk [17].

Polyphenols have been shown to improve the Lipid Transfer Protein System [18, 19] through several mechanisms. Moreover, evidence exists that polyphenol-enriched red yeast rice leads to the improvement of ACAT profile in mice, suggesting the relevant hypolipemic and anti-atherogenic activity of dietary supplementation with natural antioxidants in modulating lipoprotein assembly. Moreover, it has been demonstrated that grape polyphenols lead to functional HDL particles, enhancing reverse cholesterol transport from tissues to the liver in aged rats, this effect being accompanied by improved profile of PON1, LCAT and CETP [18].

Overall, our results, together with the modification of the apolipoprotein profile (Apo A1 and Apo B, in particular) demonstrate that BPF leads to enhanced “functional” HDL and concomitant inhibition of the tendency to develop oxidized LDL and atherogenesis. This is in agreement with our previous data showing an amelioration of both the folding of lipoprotein particles and the Lipid Transfer Protein System as well as with data collected from other groups suggesting that dietary polyphenols contribute atherogenesis prevention ameliorating the lipid profile.

## Conclusions

The mechanism through which BPF improves lipemic profile via modulation of Lipid Transfer Protein System needs to be further clarified. However, we suppose that antioxidant properties of BPF seen in vitro and in vivo may play a significant role. Indeed, in our experiments, we have shown a significant reduction of lipid peroxidation biomarkers (TBARS) in BPF-treated rats compared to the rats treated with placebo, producing a reduction of oxyLDL content, thus suggesting that anti-oxidant properties of BPF may have a crucial role in this process.

In conclusion, our data show that BPF produces hypolipemic effect in rats undergoing a fat-rich diet. This effect is associated with a more efficient Lipid Transfer Protein System, leading to functional HDLs and reduced oxyLDLs, an effect associated with antioxidant response. Overall, this is in favour of BPF dietary supplementation in hyperlipemic disease states to antagonize cardiometabolic risk.

## Abbreviations

ACAT: Acetyl-Coenzyme A acetyltransferase
AMPK: Adenosine Monophosphate-activated Protein Kinase
Apo A1: Apolipoprotein A1
Apo B: Apolipoprotein B
BPF: Bergamot Polyphenolic Fraction
CETP: Cholesteryl Ester Transfer Protein
HMGCoA reductase: Hydroxy-Methyl-Glutaryl CoA reductase
LCAT: Lecithin Cholesterol Acyltransferase
MS: Metabolic Syndrome
NAFLD: Non-Alcoholic Fatty Liver Disease
oxy-LDL: oxidized Low-Density Lipoprotein
pCEH: pancreatic Cholesterol Ester Hydrolase
PON1: Paraxonoase 1
SP1: Specificity Protein 1
SREBP2: Sterol Regulatory Element-Binding Protein 2
TBARS: Thiobarbituric Acid Reactive Substances
